# Genito-Urinary Surgery

**Published:** 1905-10-21

**Authors:** 


### Progress in Medicine and Surgery.

### GENITO-URINARY SURGERY.

**What Place has the Catheter in the Modern Treatment of Prostatic Urinary Obstruction?**—The technique of prostatectomy, whether suprapubic or perineal, has been so much improved in recent years, and the results of the operation, for that and other reasons, are now so much better than formerly, that it is incumbent upon all practitioners who have to treat the malady to earnestly reconsider the principles of treatment which were accepted as sound by almost all surgeons but a few years ago. A series of papers in a recent number of the "Annals of Surgery" [1] exhibits well various modern views upon this subject. Not many years ago the catheter was regarded as the main agent in the treatment of prostatic obstruction, but to-day, if we are to believe some writers, that instrument should be almost entirely discarded. We cannot ourselves accept this view, and we maintain that there are many cases which even to-day are more suitably treated by catheterisation than by radical operations on the prostate gland. This view of the matter is supported by Paul Thorndike in his paper among those alluded to above. Many of the victims of prostatic obstruction, as that writer points out, seek relief from the surgeon when they are very old and infirm—when, in fact, they are very bad subjects for any surgical operation, and when, therefore, only operations of necessity should be performed upon them, and prostatectomy cannot justly be regarded as necessary, because in many cases the patients can be made comfortable for the rest of their lives by the daily use of a catheter. The catheter should be used for such cases just so long as it serves to successfully palliate the symptoms; but when palliation can no longer be obtained, then the time for operation, even with its inevitable risks, will have come. At this period the use of the catheter must not be persisted with, for otherwise the failure of palliation with its accompaniments of pain, loss of sleep, and exhaustion will materially diminish the chances of success of the operation then necessary. Thorndike points to another class of case for which the radical operation is contra-indicated—namely, that in which there is atony of the bladder. If prostatectomy is performed the bladder will still remain atonic, and a catheter will have to be used after the operation. In this class of case the catheter should be regularly used, and overdistension prevented, until the bladder shows signs of returning power. The cases about which the greatest difference of opinion exists as to treatment are those in which the patients are good or fair surgical subjects, and in whom the obstructive symptoms necessitate recourse either to the catheter or to prostatectomy. If patients such as these are intelligent and cleanly, the possibilities of palliation by a routine use of the catheter and the possibilities of cure by an operation with at least a five per cent. mortality, should be fairly put before them, and it will be found that the majority will select the method of treatment with the catheter, for it is safe until it becomes difficult or irritating. In the latter event it ceases to be safe, and operation should be recommended. If patients in a similar state as to constitution and as to local conditions are ignorant, poor, and uncleanly, then the advisibility of immediate operation should be impressed upon them, for if such patients are not operated upon they are practically certain to acquire persistent cystitis, with its attendant dangers and then to have to submit to prostatectomy in the presence of very unfavourable conditions. The difference in the risks and the results of prostatectomy performed before or after the development of bladder or ureteral and kidney infections can hardly be exaggerated. Francis S. Watson points out how valuable it would be at the present time to have reported cases of prostatic obstruction treated by the catheter throughout, with special reference to the mortality and the time which elapsed between the beginning of catheter treatment and death from the secondary results of the prostatic hypertrophy or of the catheter treatment. He believes such a collection of cases would bring home to the profession at large the dangers attending catheter treatment, and that the contrast between the results of the above treatment and of prosatectomy would lead to the selection of the radical operative treatment at an early period in the malady. He is convinced that prostatectomy practised at an early stage in the disease would prove far less fatal than treatment by catheter. L. S. Pilcher writes that the catheter may certainly be used as a temporary resort, but, in the light of present experience, always rather under protest than as a measure possessing the full recommendation of the surgeon. It is obvious that if prostatectomy has been successfully performed, and the patient has in consequence reacquired the power of normal micturition and does not suffer from any of those untoward consequences of prostatectomy which are sometimes met with, he is in a much more satisfactory position than a patient who is dependent upon a catheter. If all the surgeons who have to treat these cases could get results equal to those recorded by those who are specially expert in performing the operation, then prostatectomy could be recommended more strongly than it can be at present. The published statistics, we are convinced, show prostatectomy in too favourable a light. Besides its considerable mortality, there are various possible untoward results such as imperfect recovery, incontinence, stricture formation, fistula, etc., and these have to be considered in estimating the relative advantages or disadvantages of prostatectomy and catheterisation. We shall shortly return to a discussion of these drawbacks of prostatectomy.

[1] Annals of Surg., April 1905.

